# Knockdown of DNA methyltransferase-1 inhibits proliferation and derepresses tumor suppressor genes in myeloma cells

**DOI:** 10.3892/ol.2014.2481

**Published:** 2014-08-26

**Authors:** WENWEN ZHOU, HUYING CHEN, XIULI HONG, XIAOQING NIU, QUANYI LU

**Affiliations:** Department of Hematology, Zhongshan Hospital, Xiamen University, Xiamen, Fujian 361000, P.R. China

**Keywords:** DNA methyltransferases, siRNA, tumor suppressor gene, myeloma cells

## Abstract

DNA methyltransferases (including DNMT1, DNMT3A and DNMT3B), catalyze the transfer of methyl groups from S-adenosyl-l-methionine to cytosine position 5; this methylation in promoter regions silences gene expression. In addition, DNMT1 plays a critical role in the maintenance of genomic DNA methylation during DNA replication. In the present study, silencing of *DNMT1* with siRNA was performed in RPMI-8226 human multiple myeloma (MM) cells, and the impact on gene methylation status and proliferation of the cells was analyzed. Upon *DNMT1* downregulation, proliferation decreased significantly compared with that in the control, non-transfected cells. The expression of B-cell lymphoma 2 and nuclear factor κB proteins was also significantly reduced. Furthermore, nested methylation-specific polymerase chain reaction revealed that methylation of the tumor suppressor genes, suppressor of cytokine signaling 1 and *p16,* was significantly reduced upon DNMT1 knockdown. Our results suggest that *DNMT1* silencing may be a promising strategy to consider during development of novel MM treatment strategies.

## Introduction

Multiple myeloma (MM) is a malignant hematological disease characterized by the accumulation of clonal plasma cells and the presence of monoclonal immunoglobulin in blood, osteolytic lesions, hypercalcemia and immunodeficiency. It accounts for approximately 1% of all cancers and 10–15% of hematologic malignancies (1,2). In recent years, the molecular and clinical knowledge emerging from studies of MM pathogenesis and the response to treatments has grown exponentially. This has facilitated the generation of new drugs and clinical strategies that have significantly improved the prognosis of certain patients with MM. However, in the long term, MM remains an incurable disease. Patients with acquired drug resistance invariably relapse, and salvage therapy is not effective (3,4).

Methylation of DNA is one of the most important modifications of the mammalian genome, DNA methylation is achieved by a family of DNA methyltransferase enzymes (DNMTs) that transfer the methyl group from the donor S-adenosyl methionine to the fifth carbon of cytosine. Aberrant DNA methylation is a common epigenetic mechanism implicated in the etiology of numerous human cancers. Hypermethylation of a number of tumor suppressor genes occurs at CpG islands in the promoter, leading to gene inactivation (5–8). Previous Studies have shown that hypermethylation of genes encoding cell cycle inhibitors p15 and p16, the apoptosis regulator death-associated protein kinase, the tumor suppressor Ras association domain-containing protein 1 and suppressor of cytokine signaling 1 (SOCS1) occurs frequently in MM patients (9–14).

In the current study, RNA interference was employed to knock down *DNMT1* expression in human MM cells to investigate the association between DNMT1 expression and the proliferative activity, tumor suppressor gene expression and gene methylation levels of myeloma cells.

## Materials and methods

### Cell culture and experimental reagents

The RPMI-8226 human MM cell line was obtained from the Cell Bank of the Chinese Academy of Sciences (Shanghai, China) and was cultured in RPMI-1640 (Gibco-BRL, Grand Island, NY, USA) supplemented with 10% fetal bovine serum (Gibco-BRL) in a 5% CO_2_ atmosphere at 37°C. Lipofectamine 2000 was purchased from Invitrogen Life Technologies (Carlsbad, CA, USA). RevertAid First Strand cDNA synthesis kit and DreamTaq Green PCR master mix were from Fermentas (Glen Burnie, MD, USA). QIAamp DNA mini kit and EpiTect Bisulfite kit were from Qiagen (Hilden, Germany). Cell Counting Kit-8 (CCK-8) and Cell Cycle Analysis kit were from MultiSciences Biotech (Hangzhou, China).

### siRNA transfection

Recombinant plasmids containing the green fluorescent protein (GFP) gene which expresses GFP, and the transfection efficiency may be directly observed under an inverted fluorescence microscope (CKX41-F32FL, Olympus, Tokyo, Japan). RPMI-8226 cells were seeded in six-well plates overnight and then transfected with siRNA or negative control siRNA oligonucleotides (containing the GFP gene which emits green light) that were precomplexed with Lipofectamine 2000 (Invitrogen Life Technologies). The medium was refreshed after 6 h with complete growth medium, and the cells were incubated for an additional 48 h. Following this, antibiotic selection (0.2 μg/ml puromycin; Invitrogen Life Technolgies) was initiated and continued for 14–20 days prior to selection of stably transfected cells. The siRNA sequences used to target DNMT1 were 5′-CACTGGTTCTGCGCTGGGA-3′ (sense) and 5′-AAGTCTTCTGACGCTGCTGCCTGGTCCAG-3′ (antisense), and were designed based on GenBank accession no. NM_001379.1.

### Quantification of proliferation using the CCK-8 assay

Cells transfected with DNMT1 siRNA or siRNA control were incubated for 1–5 days at a density of 1×10^3^ cells/well in 96-well plates. CCK-8 (10 μl) was added in each well, followed by an additional 3-h incubation prior to reading the absorbance at 450 nm using a microplate reader (Bio-Rad 680; Bio-Rad, Hercules, CA, USA). An average value from three wells was obtained for each group of RPMI-8226 cells to plot the growth curve.

### Cell cycle assays

Transfected cells were seeded at a density of 1×10^6^ cells/well in six-well plates. After a 48-h incubation, the cells were collected and washed twice with ice-cold phosphate-buffered saline (PBS), fixed in 70% ethanol at room temperature (RT) for at least 30 min, and stored at −20°C overnight. For analysis, the cells were washed twice with PBS, stained with propidium iodide (10 μg/ml; MP Biomedicals, Santa Ana, CA, USA) and incubated at 37°C for 30 min. Fluorescence was measured with a flow cytometer (BD Bioscience, San Jose, CA, USA), and the data were analyzed using Cell ModFit software (BD Bioscience). The experiments were performed three times in order to derive a mean value.

### Western blot analysis

RPMI-8226 cells were incubated at 4°C for 30 min in lysis buffer. Protein concentration was determined with the Bio-Rad protein assay system (Bio-Rad). Equal amounts of protein lysates (50 μg) were analyzed by performing SDS-PAGE and electrotransfer of proteins to polyvinylidene fluoride membranes. Membranes were washed with 1× Tris-buffered saline with Tween-20 (TBST), blocked for 1 h at RT in skimmed milk/TBST, and then immunoblotted with the appropriate primary antibodies. The primary antibodies included monoclonal mouse anti-human DNMT1, monoclonal rabbit anti-human B-cell lymphoma 2 (BCL2), monoclonal rabbit anti-human nuclear factor κB (NF-κB) (Abcam, Cambridge, UK), and monoclonal mouse anti-human β-actin (Santa Cruz Biotechnology, Inc., Dallas, TX, USA). The β-actin was used as a loading control. The next day, the membranes were washed with 1× TBST and incubated with anti-rabbit or anti-mouse IgG horseradish peroxidase-conjugated secondary antibodies diluted to 1:3000 in skimmed milk/TBST for 1 h at RT. Proteins were visualized using a Vazyme E411-01 enhanced chemiluminescence detection kit [Vazyme Biotech (Nanjing) Co., Ltd., Nanjing, China].

### Quantitative polymerase chain reaction (qPCR) assays

Total RNA was isolated from transfected cells using TRIzol reagent (Invitrogen Life Technologies). cDNA was synthesized using the RevertAid First Strand cDNA synthesis kit (Fermentas) according to the manufacturer’s instructions. The primers used for amplifying *DNMT1* were 5′-ACCATCACATCTCATTTTGC-3′ (sense) and 5′-GGTTTGACTTCGGAGTCTCT-3′ (antisense). The primers used for amplifying β-actin were 5′-GTGGGGCGCCCCAGGCACCA-3′ (sense) and 5′-CTCCTTAATGTCACGCACGATTT-3′ (antisense). qPCR was carried out with DreamTaq Green PCR master mix (Fermentas), and the analysis was performed in an Eppendorf PCR device 00135 (Eppendorf, Hamburg, Germany). The thermal cycling was as follows: 94°C for 3 min; 30 cycles of denaturation at 94°C for 30 sec, annealing at 55°C for 30 sec and extension at 72°C for 1 min; and a final 10-min extension at 72°C. The relative quantity was analyzed with the 2^−ΔΔCt^ method. β-actin mRNA was used as a control, and each experiment was performed in triplicate.

### Nested methylation-specific PCR assays

Genomic DNA was extracted from transfected cells with a QIAamp DNA mini kit (Qiagen) and was subjected to bisulfite modification with EpiTect Bisulfite kit (Qiagen) according to the manufacturer’s instructions. The stage-1 PCR products were diluted 50-fold, and 5 μl of the product was subjected to a stage-2 PCR, in which primers specific to methylated or unmethylated template were used. Primer sequences used in the stage-1 amplification of the *SOCS1* and *p16* genes are as follows: SOCS1 sense, 5′-AACTGCTTTTTCGCCCTTAGC-3′ and SOCS1 antisense, 5′-CAGCTCGAAGAGGCAGTCG-3′; p16 sense, 5′-GAAGAAAGAGGAGGGGTTGG-3′ and p16 antisense 5′-CTACAAACCCTCTACCCACC-3′. The PCR amplification protocol for stage 1 was as follows: 95°C for 3 min; 40 cycles of denaturation at 95°C for 30 sec, annealing at 60°C for 30 sec and extension at 72°C for 30 sec; and a final 10-min extension at 72°C. In the stage-2 PCR, annealing temperatures were increased to 65°C for *p16*, and annealing times were increased to 45 sec for *SOCS1*. All assays were conducted in triplicate.

### Statistical analysis

Data are presented as the mean ± standard deviation. Statistical analysis was performed using Student’s t-test or analysis of variance. P<0.05 was considered to indicate a statistically significant difference. GraphPad Prism 5.0 software (GraphPad Software Inc., La Jolla, CA, USA) was used for statistical analysis.

## Results

### Effective downregulation of DNMT1 expression in human RPMI-8226 cells by DNMT1 siRNA

**Figure 1:**
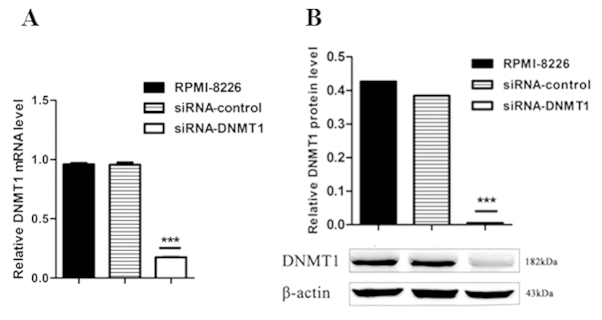
Downregulation of DNMT1 mRNA and protein expression by siRNA in human RPMI-8226 cells. (A) Quantitative polymerase chain reaction was used to analyze DNMT1 mRNA expression. (B) Western blot analysis of DNMT1 protein expression. The data are from three independent experiments, ^***^P<0.0001, compared with the negative control group and the non-transfected control group. DNMT1, DNA methyltransferase 1.

The recombinant plasmid of siRNA targeted against DNMT1 was constructed and successfully transfected into RPMI-8226 cells. The transfected RPMI-8226 cells were visible (emitted green light) under an inverted fluorescence microscope (CKX41-F32FL; Olympus Corporation). Compared with the two control groups, the DNMT1 expression was decreased significantly, both at the mRNA (Fig. 1A) and protein (Fig. 1B) levels (P<0.001), as determined by qPCR and western blot analysis, respectively. This confirmed the transfection of siRNA to the genome and its stable expression.

### DNMT1 silencing inhibits the proliferation capacity of human RPMI-8226 cells

**Figure 2:**
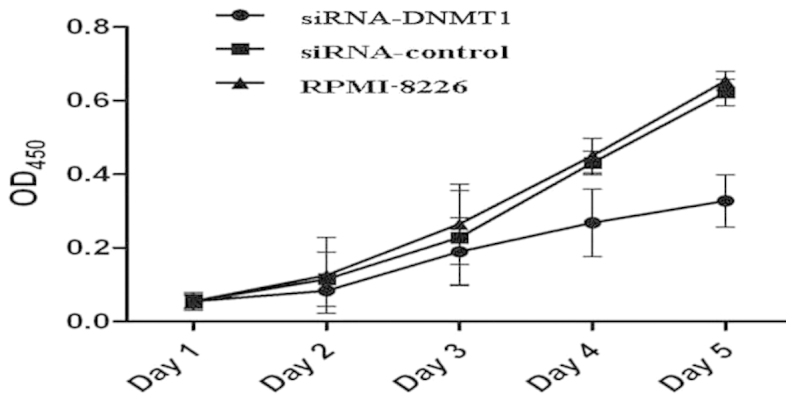
Effect of DNMT1 silencing on RPMI-8226 cell growth. Cell growth was evaluated using the Cell Counting Kit-8 assay. After 5 days of transfection, the growth rate of the DNMT1 siRNA group was significantly lower than that of the negative control group and the non-transfected control group (P<0.01). DNMT1, DNA methyltransferase 1

The effect of *DNMT1* silencing on cell proliferation was determined by a CCK-8 assay. As shown in Fig. 2, it was found that the *in vitro* cell growth rate of the *DNMT1* siRNA group was significantly lower than that of the other two groups 5 days after siRNA transfection (P<0.01), while no significant difference was found between the negative control group and the non-transfection group (P>0.05).

### Effect of DNMT1 siRNA on the cell cycle of RPMI-8226 cells

**Figure 3:**
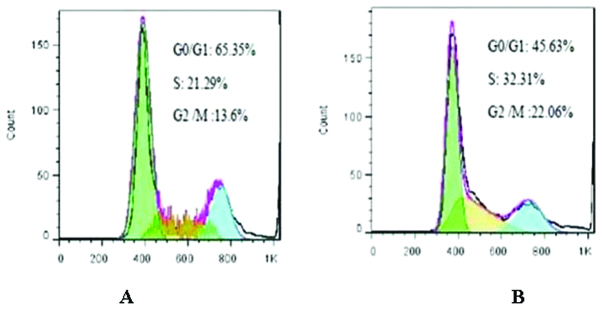
Effect of DNMT1 knockdown on the cell cycle. Cells were subjected to propidium iodide staining and flow cytometry analysis. Compared with the untransfected cells, treatment with DNMT1 siRNA increased the number of cells in the G0/G1 phase, while reducing the number of cells in S and G2/M phases. (A) siRNA-DNMT1 and (B) RPMI-8226 groups. All the experiments measuring cell cycle were repeated three times. DNMT1, DNA methyltransferase 1

Cell cycle analysis showed that compared with untransfected cells, treatment with *DNMT1* siRNA increased the number of cells in the G0/G1 phase (65.35±0.08 vs. 45.63±1.10%, P<0.05), while reducing the number of cells in the S and G2/M phases (S stage: 21.29±1.54 vs. 32.31±0.72%, P<0.05; G2/M stage: 13.6±1.03 vs. 22.06±0.66%, P<0.05) (Fig. 3). The percentage of each phase of cells in the siRNA-control group was similar to that of the RPMI-8226 group (P>0.05). The percentage of each phase of cells was not considered to be statistically significant between the negative control group and the RPMI-8226 group (P>0.05).

### DNMT1 siRNA reduces NF-κB and Bcl-2 protein expression in human RPMI-8226 cells

**Figure 4:**
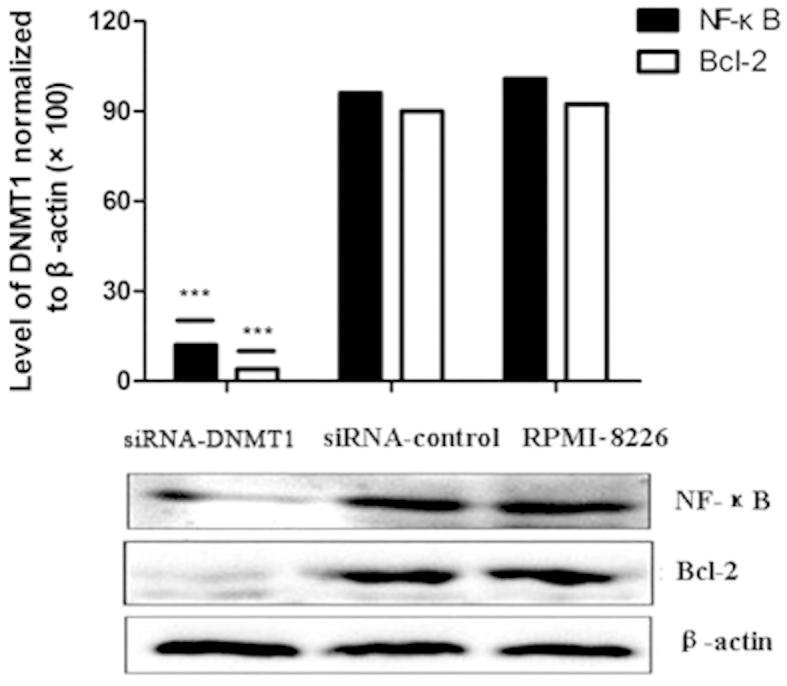
DNMT1 siRNA reduces NF-κB protein and BCL2 protein expression in RPMI-8226 cells. Compared with the negative control group and the non-transfected control group, NF-κB and BCL2 expression were both significantly reduced in the DNMT1 siRNA group (P<0.05). β-Actin was the internal control. DNMT1, DNA methyltransferase 1; NF-κB, nuclear factor κB; BCL2, B-cell lymphoma 2.

RPMI-8226 cells were grown and transfected with *DNMT1* siRNA or negative control siRNA oligonucleotides. Protein expression was detected by western blot analysis. The results suggested that compared with the negative control group and the untransfected group, both NF-κB and Bcl-2 protein expression was significantly reduced in the *DNMT1* siRNA group (P<0.05, Fig. 4).

### DNMT1 siRNA induces demethylation of the tumor suppressor genes SOCS1 and p16

**Figure 5:**
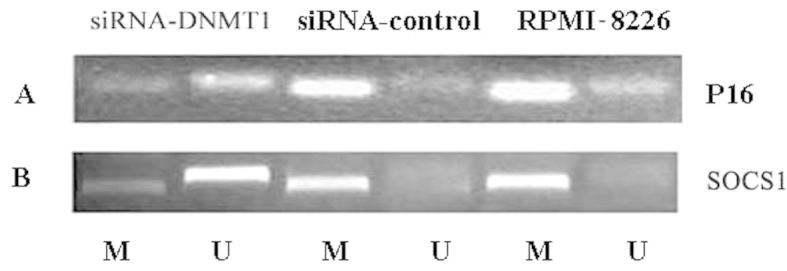
Demethylation of *SOCS1* and *p16* genes upon treatment with *DNMT1* siRNA. RPMI-8226 cells were grown and transfected with *DNMT1* siRNA or negative control oligonucleotides. Genomic DNA from the cells was isolated and subjected to nested methylation-specific polymerase chain reaction assays. All experiments were performed three times. (A) *p16* gene. (B) *SOCS1* gene. *SOCS1*,suppressor of cytokine signaling 1; DNMT1, DNA methyltransferase 1; M, methylated; U, unmethylated.

In human RPMI-8226 cells, the tumor suppressor genes *SOCS1* and *p16* were identified to be highly methylated by nested methylation-specific PCR (Fig. 5). Following transfection of RPMI-8226 cells with *DNMT1* siRNA, *SOCS1* and *p16* genes lost methylation marks, showing that the high-level methylation at these loci can in part be reversed (Fig. 5).

## Discussion

DNA methyltransferases, including DNMT1, DNMT3A and DNMT3B, catalyze the methylation of human genomic DNA. Methylation of DNA at C-5 of cytosine plays a key role in the regulation of human genes and can result in X-chromosome inactivation, genomic imprinting and silencing of proviral elements and retrotransposons (15). Aberrant methylation, particularly in the promoter regions of tumor suppressor genes, alters gene expression and can facilitate human tumorigenesis. DNMT1, the major DNMT in adult cells, preferentially acts on hemimethylated CpG substrates and is involved in the maintenance of genomic DNA methylation during DNA replication (16,17).

Aberrant methylation in the promoter regions of tumor suppressor genes is implicated in the pathogenesis of numerous types of malignant tumors. Thus, epigenetic therapies incorporating DNMT inhibitors are expected to induced demethylation, re-expression, and functional recovery of silenced tumor suppressor genes (18–20). Both *SOCS1* and *p16* are commonly silenced in malignant tumors, and are also silenced by methylation in familial MM (21–23).

In the present study, it was found that DNMT1 expression was significantly downregulated at both the mRNA and protein level in RPMI-8226 myeloma cells following treatment with *DNMT1* siRNA. The downregulation of DNMT1 expression inhibited cell growth, most likely by inducing arrest at the G0/G1 phase. Our results also showed that upon *DNMT1* silencing, the methylation of the *SOCS1* and *p16* promoters was reduced, and the genes were re-expressed. These observations suggest that demethylation of tumor suppressor gene promoters should be further evaluated as a therapeutic strategy in myeloma.

Tumor suppressor gene inactivation has previously been correlated with DNMT1 overexpression in various types of cancer, including hematological malignancies (24–27). Robert *et al* (28) reported that siRNA-mediated *DNMT1* silencing can trigger reactivation of tumor suppressor gene expression and function in HCT116 colon cancer cells. This is consistent with the findings of the present study that inactivation of *SOCS1* and *p16* may be associated with DNMT1 overexpression. By contrast, Ting *et al* reported that single *DNMT1* gene knockout was insufficient to trigger demethylation of tumor suppressor gene promoters and restore their function (29). This highlights the fact that mechanisms that control the expression and inactivation of tumor suppressor genes vary across different tumor cells.

NF-κB is an important transcription factor involved in transcriptional regulation of various genes that in turn modulate immune cell activation, apoptosis and differentiation processes. Sustained activation of NF-κB plays an important role in the pathogenesis of MM. For example, NF-κB activation can enhance the expression of adhesion molecules, promoting homing of myeloma precursors and the production of tumor cell growth factors. NF-κB can also promote the secretion of interleukin 6 by adhesion with extracellular matrix proteins and bone marrow stromal cells, initiating various signal transduction pathways and promoting tumor cell proliferation and drug resistance (30,31). Therefore, inhibition of NF-κB overexpression is an effective way to induce apoptosis and overcome the drug resistance associated with certain MM cells. Indeed, NF-κB is now one of the key therapeutic targets in MM (32,33).

BCL2 is a critical pro-survival member of the BH domain-containing superfamily. BCL2 inhibits apoptosis and is involved in the pathogenesis of a variety of hematological tumors, including MM (34). Specifically, overexpression of BCL2 protein is associated with the survival and drug resistance of MM cells (35).

In the present study, compared with the untransfected and negative control groups, the expression of NF-κB and BCL2 proteins was significantly reduced upon *DNMT1* knockdown. Although the precise mechanism for this remains unclear, these data suggest that targeting DNA methylases may lead to downregulation of critical tumor survival factors, thereby inducing tumor cell death. In summary, the results of the present study provide key evidence that targeting methylases, either by RNA-mediated knockdown approaches or through the use of small molecules, may be an effective means of inducing tumor regression. The challenge will be to selectively de-repress only those gene targets responsible for tumor survival and to avoid de-repression of genes in neighboring normal cells, as this could have deleterious effects.
